# Data Collection Schemes for Animal Monitoring Using WSNs-Assisted by UAVs: WSNs-Oriented or UAV-Oriented

**DOI:** 10.3390/s20010262

**Published:** 2020-01-02

**Authors:** Rodolfo Vera-Amaro, Mario Eduardo Rivero-Ángeles, Alberto Luviano-Juárez

**Affiliations:** 1 Instituto Politécnico Nacional—(SEPI-UPIITA-IPN), Mexico City 07740, Mexico; 2 Instituto Politécnico Nacional—(CIC-IPN), Mexico City 07738, Mexico

**Keywords:** WSNs, UAVs, clustering, data collection schemes, energy consumption

## Abstract

Wireless sensor networks (WSNs) and unmanned aerial vehicles (UAVs) have been used for monitoring animals but when their habitats have difficult access and are areas of a large expanse, remote monitoring by classic techniques becomes a difficult task. The use of traditional WSNs requires a restrictive number of hops in a multi-hoping routing scheme, traveling long distances to the sink node where data is stored by nodes and UAVs are used to collect data by visiting each node. However, the use of UAVs is not straightforward since the energy balance between the WSN and UAV has to be carefully calibrated. Building on this, we propose two data collection schemes in clustered based WSNs: (1) WSN oriented and (2) UAV oriented. In the former, nodes within each cluster member (CM), send information to their cluster head (CH) and for recollection, the UAV visits all CHs. As the UAV visits many CHs the flight time is increased. In the latter, all CHs send data from their CMs to a sink node, hence, the UAV only visits this node, reducing the flying time but with a higher system energy cost. To find the most suitable scheme for different monitoring conditions in terms of the average energy consumption and the buffer capacity of the system, we develop a mathematical model that considers both the dynamics of the WSN along with the UAV.

## 1. Introduction

Wireless sensor networks (WSNs) have been evolving since the internet, communication, and information technologies have been converging as the size of commercial sensor nodes have shrunk while increasing the processing capacity and consuming less energy. Using WSNs has very important advantages over traditional monitoring techniques in different sectors of the society like the military, industrial, civil, and nature among others. WSNs are composed of many nodes communicating among them to convey relevant information for a particular application that defines the requirements and specifications that they must have [1]. These WSNs have been increasingly used in cases where human access is difficult or the costs are significantly expensive for example, in environmental, vegetation, farming, or animal monitoring. In these specific applications, sensor nodes with limited processing and memory capacity, small, cheap, low energy consumption, and long lifetime are usually used and can be deployed in a certain area for different purposes [2,3]. Though several studies and improvements on WSNs have been made, there are still many issues to solve, primarily energy consumption reduction and data accuracy. It is expected that sensor nodes provide a large battery operation time in areas of difficult access but it is also necessary to solve the aforementioned issues with alternative techniques, methods, strategies, or frameworks involving the operation and logistics of the monitoring process.

A WSN is usually formed with sensor nodes and gatherer nodes that collect data from the sensor nodes. Gatherer nodes can take the role of a base station (BS), but if this BS is in a remote location, the network can be assisted by a mobile device to carry the information to the BS. In this work, according to the monitoring application, we focus our study on a star topology in which the BS is at the corner and outside of the observation area and the sensor nodes are randomly placed inside the observation area. Usually, the BS has a higher processing capacity than regular nodes and can be used as a gateway to send data over the internet, or over some other communication systems [4]. In order to reduce energy in the system, an emergent alternative is the use of UAVs as mobile collecting nodes, to bring the information to the base station. The use of aerial devices to collect sensor data has many advantages compared to the traditional schemes, such as: (a) Multi-hop or cluster routing with one or more static sink nodes, in terms of energy consumption [5], (b) being able to access locations with difficult and risky access by people or for land vehicles, or (c) in cases where it is expensive and impractical to rent manned aerial vehicles (MANs) [6].

Recently, the use of drones has expanded because their costs are affordable, and their variety of types, sizes, and features have been increasing, which has led to a wide set of uses from military, civil, to domestic applications. It has been shown that UAVs as data collection nodes have improved the energy consumption performance of WSNs, such as increasing the efficiency of data transmission and its lifetime, as well as to collect and transmit information stored in a network of ground-to-air communication [7].

Studies on monitoring with WSNs and UAVs of wildlife, and in particular the collection of data from sensors in endangered species has been increasing in recent years. In particular, these schemes are used to monitor endangered species like ocelots from Barro Colorado [8] or mangabey monkeys from Uganda [9] among others. Although the importance of the applications and energy consumption aspect, the related bibliography is not extensive [10]. Hence this work focuses on monitoring endangered animals in large extensive regions, where it is not easy to implement and operate a WSN due to environmental conditions and where it is difficult to replace the node’s batteries.

**Figure 1:**
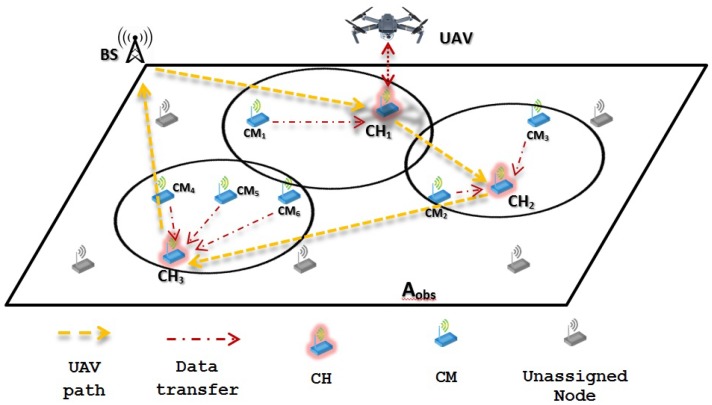
WSN (wireless sensor networks) oriented scheme scenario example.

**Figure 2:**
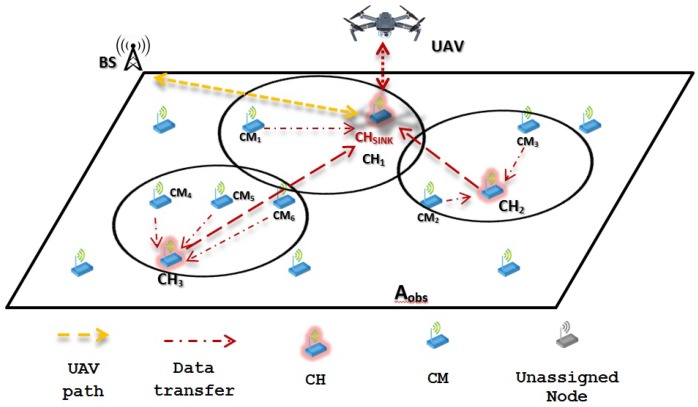
WSN-oriented scheme scenario example.

Building on this, two main recollecting data schemes are proposed: (1) WSN-O, and (2) (unmanned aerial vehicles) UAV-O. In both schemes, data is obtained when the monitored animal moves close to the static sensor nodes that form the WSN. This can be done by using specific sensors, such as movement detection, heat detection, image processing, among others. Additionally, information regarding the animals can be done by placing RF (radio frequency) transmitters in the animal’s bodies, e.g., neck, legs, or wings. The performance of these schemes are studied in terms of average energy consumption. The main ideas of the proposed schemes are:

1 WSN-O: In this scheme, the main purpose is to reduce energy consumption in the WSN. As such, after the clusters are formed, data is stored at the cluster head (CH) nodes, effectively reducing energy consumption form these nodes since no further packet transmissions are made to the sink node. However, the drone consumes more energy in the data collection procedure since it has to visit all CHs, enlarging its trajectory. This is depicted in Figure 1, where the yellow dotted line represents the flight path of the UAV, visiting all the CHs and the red thin dotted line the data transfer from the CMs (cluster members) to the CHs and the red bold dotted line the recollecting data of the UAV from each of the CHs;
2 UAV-O: In this scheme, energy consumption is increased in the WSN by concentrating the data at a single point in the monitored area. As such, multiple transmissions have to be made form the CHs to this specific sensor. In contrast, energy consumption is reduced at the drone since its trajectory is much shorter because, to recollect information, it only travels to a single destination in the system. In Figure 2, it can be noticed that the flight path is only to this single node where the CHs are sending their data represented with the red bold dotted.

To the best of the author’s knowledge, this is the first work to study in-depth the energy reduction schemes at either the assisted WSN by UAVs or at the UAVs acting as data collectors in a WSN. Indeed, in previous works, the available energy in sensors and/or the UAV is assumed to be infinite. Conversely, in this work, it is assumed that, in the WSN-O approach, the drone has enough energy to perform long-time flights in order to collect data from the network while the energy at the WSN is rather limited. In the UAV-O scheme, it is assumed that the WSN has enough energy to perform high energy-consuming transmissions for the sake of allowing the drone to reach a single point, effectively considering a limited energy supply at the UAV. However, the limits of these schemes are not usually given. Specifically, this article aims at answering the following questions:

(a) Is there a threshold monitored area where one scheme performs better than the other?
(b) Is there a specific number of required nodes for these schemes to perform adequately?
(c) Does one of these schemes always entail a better performance over the other?
(d) Are both schemes suitable in different system conditions?

To this end, it is given a mathematical analysis based on Markov Chains to calculate the average energy consumption in the system considering, both, the WSN and UAV for a different number of nodes and a monitored area. Additionally, a consumption energy mathematical model for the UAV flight paths based on the backstepping and approximate linearization methods is proposed and developed to achieve accurate values that can be used in real scenarios, including the path flight, type of UAV, battery capacity, vertical and horizontal velocity, altitude, and time flight. Hence, the results presented in this work can give clear guidelines for the use of drones in assisting WSNs applications.

Thus, the main contributions of this paper are as follows:

- Two energy reduction schemes for WSN assisted by UAVs are proposed, investigated, and modeled;
- A general analytical framework is developed to calculate the average energy consumption in the system to evaluate the performance of WSN assisted by UAVs, considering different system variables;
- Practical guidelines for the system design are provided. As such, by knowing the main system operation parameters such as observation area, the network administrator can select the appropriate collecting scheme (WSN-O or UAV-O) and define the system variables, such as, number of nodes, initial energy, and system lifetime;
- A consumption energy model is developed for the UAV given a specific flight path plan.

This article is organized as follows: First, some related works are analyzed and discussed. Then the analysis and development of the WSN to UAV system model for tracking animals in large extension areas are presented. After describing the scenario, the case study and operation hypotheses are proposed. The design and implementation of the mathematical model are then presented, including some numerical simulations as well as the validation results, carried out with the random route. Next, the proposed mathematical model for the UAV flight paths and total system is developed to calculate the total energy consumption and number of packets transmitted. After this, some numerical results of the simulation and analytical model are presented together with its corresponding assessment for different scenarios. Finally, some concluding remarks are provided.

## 2. Related Work

Recent works show the advantages of using mobile sink nodes to gather information from WSNs such as being able to reduce the traffic load in the network and improve energy efficiency as well as the lifetime of the network. For instance, in [11], the authors propose a new routing algorithm for a mobile sink node for collision evasion in an environment with obstacles, which collects information from a WSN-based routing cluster. According to this routing method, the nodes selected as CHs collect data from their respective CMs and then transmit this information to the mobile sink node. The mobile node begins its route periodically from the initial site to each of the cluster headers defined by the Cluster Hierarchy Adaptive Low Energy protocol (LEACH), with a single-hop method and finally returns to the initial site. Due to the problem of the scheduled flight, a programming algorithm based on coverage charts is proposed to detect possible collisions. Their proposed energy consumption model is only for the sensor nodes, assuming unlimited energy consumption of the mobile node sink.

According to works such as [4,12,13,14,15,16,17,18,19,20,21], there are many applications that use WSNs and UAVs. In these works, the usual technique to collect data from the sensor node is directly towards the BS because nodes can be very simple with no major processing tasks and the implementation can be faster but with inefficient energy consumption and limited coverage. Collecting the information by cluster routing [22] is another commonly used model. In this configuration, a single or multiple groups of sensors, also called clusters, are formed and each of the clusters has a leading group sensor or also called, the cluster head (CH). Each of the CHs collects the information from their respective sensor nodes, also called cluster members (CMs), and transmits it to the other neighboring CH, or even to the BS (if it is within reach). Hence, this cluster strategy allows the energy consumption and packet rate to be more efficient while covering larger distances. Although in both cases we have corresponding advantages, a range of large coverage is required by the BS. Neighbor nodes need to be placed relatively close to each other among clusters in WSN cluster-based and due to large areas, such as the endangered animal monitoring, a high number of nodes would be required, which could result in higher implementation costs. Additionally, a problem that is known as hot-spot [23,24] could be present, which is that when nodes cannot directly reach the BS, the nodes closer to the collecting node or CHs deplete their battery faster, affecting both the coverage of the node and lifetime of the WSN.

The work in [25] proposes an energy management in a WSN with a multiple sinks (EMMS) algorithm to streamline the energy consumption of the WSN using multiple mobile sink nodes. The challenge of the proposed protocol design is to balance the payload between sink mobile nodes and the energy consumed between the sensor nodes through the flight control of each mobile sink node and, finally, it compares the efficiency with similar algorithms (SDMA [26] and DAWN [27]). The drawbacks of this proposal are the economic cost of deploying multiple mobile nodes, the high processing operations at the sink nodes, and the complexity of the synchronization among them.

The study presented in [28] also deals with the problem of improving the delivery of data through distance priority, trying to avoid the communications in a straight line with the virtual algorithm grid-based on dynamic routes adjustment (VGDRA) as well as minimizing the cost of rebuilding routes to sensor nodes, randomly deployed, keeping optimal routes close to the last location of the mobile sink node. The rules for the reconstruction of routes are proposed requiring a limited number of nodes to carry out this process. The sensor field is divided into a virtual mesh containing cells of the same size and the nodes near the center are chosen as the cell head nodes. The mobile sink moves through the sensor field and collects the sensing data by communication with each cell head node and to reduce the average cost of communication, the path reconstruction only includes a subset of cell head nodes. The difference with the presented work is that this proposal focuses on the energy saving of the system with intelligent adjustments of the drone routes. The authors in [29] propose a mixed and complete programming approach to the base station to mitigate the energy dissipation of the WSN by introducing a mobile base station. There are several patterns of mobility of the mobile base station, concluding that the pattern of trajectory Gaussian and spiral give the best result to extend the life of the network.

It should be noted that they are independent trajectories of the information of the nodes. As in the previous work, [30] tries to calculate a defined trajectory for the mobile node sink with limited speed by means of the events’ information obtained from groups of sensors in a specific time where the mobile sink nodes are used. This is why it proposes a convex optimization model inspired by a technique of vector regression support to determine the optimal trajectory of the mobile sink without considering, unlike previous studies, predefined structures such as mesh or virtual grid or Rendezvous points. In the same way as [28], this work tries to study the generation of better routes for the mobile nodes and visit the corresponding sensors, optimizing the energy consumption of the sensor network. The proposal in [31] focuses the problem on minimizing the length of the tour or route for the collection of data utilizing the communication of the node sink to sensor node by single-hop. The algorithm is presented to plan the tour of the sink node or called here, collector-M. It is compared with an approximate linear coverage algorithm, resulting in a great decrease in the distance of M-collectors in motion for small networks. Thus, this scheme can prolong the life of the network compared to a static sink. And in the end, several M-collectors are also proposed, each one with a trajectory calculation using the aforementioned algorithm. In [32], the SinkTrail algorithm is proposed to improve the power consumption and data collection of the static sensor nodes, deployed in random positions, by the mobile node sink. The mobile sink node travels continuously at low speed and collects data, emitting control messages in specific locations, in low frequency, called footprints of the mobile sink. If these footprints are considered as virtual markers, the sensor nodes can easily identify their distances, in jump count, to these markers. Thus, it is possible to calculate the distance of each sensor to a coordinating sensor node (or cluster header) which was defined by the logical space built by the mobile node sink based on its footprints. Having defined the position of the coordinating node with respect to the sensor node and its location without the need for any geographic localization technology, such as GPS. In this way, each sensor node selects “selfishly” its next jump, according to the logical path shorter towards the coordinating node. In [33] and [34] the author considers a hybrid WSN composed of static sensors, which can only census an event attribute, while mobile nodes can evaluate multiple event attributes. According to the data received by the static sensors, the mobile sensors move to specific locations or hot spots defined by the proposed algorithm based on two phases. The former is to define the problem of programming the path of the mobile node, proposing it as a multi-round sensor and multi-attribute (MAM sensor). The second is to propose an expanded tree construction algorithm based on unallocated hot-spots for the displacement of MAM sensors. This work, unlike the proposal, needs the static sensors feedback to create the routes for the mobile sensors.

In [35], the authors propose and develop a method to collect the distributed data of a WSN concerning the consumption of energy when the network is composed of a large number of sensor nodes in agriculture applications. The proposed solution is to use UAVs as sinks mobile nodes, and Bluetooth Low Energy (BLE) for communication between the drone and sensor nodes, via a single-hop transmission. The problem found in the analysis is the distance of coverage between the drone and nodes because the BLE lacks good coverage and this manuscript does not develop a mathematical model or system simulation to predict the energy consumption in specific scenarios as proposed here.

The authors in [36,37] focus on using UAV and sensors in a cooperative way for disaster management applications. Possible solutions are researched and studied to maintain the connection between the system networks and UAVs besides the security and energy issues. Furthermore, they present a number of issues and challenges that still remain unsolved, hence, these works allow one to comprehend a much better use of WSNs with UAVs and their related advantages and problems, not only in applications as management disaster, but in many others.

Gong [38] considers that a single UAV collects data from a set of sensors on a straight line. The UAV can either cruise or hover while communicating with the sensors. The purpose is to minimize the flight time of the UAV while each sensor uploads a certain amount of data consuming a given amount of energy. They show that for multiple nodes, the flight time minimization problem can be studied as a dynamic programming (DP) problem, which reduces the case of many nodes, to a single sensor node. Most recent studies like in [39] provide an energy-efficient data gathering with a deadline for wireless sensor networks using the UAV and a series of virtual grid points, named Virtual Grid Energy Efficient Deadline Based Data Gathering (VGEEDDG), to determine the optimal virtual grid points and optimal sojourn time for deploying multiple UAVs with a minimum time required in a predetermined deadline time to collect buffer data from CHs. This deadline time for collecting data sometimes is not enough, and a single UAV cannot collect data from cluster heads with minimal energy thus, this work presents seven strategies for solving the problem of inadequate deadline time using multiple UAVs for deadline-based WSN applications. We are not focusing on the construction of a virtual grid point where multiple UAVs flight collect data from CHs with efficient consumption energy, but in pre-established routes where the CHs location is known.

One of the problems in monitoring animals with UAVs and WSNs is the path that followed is not deterministic but probabilistic analysis and can be done to approximately predict their *random walk* like in [40]. In [41], they define a specific scenario for the monitoring of zebras in an observation area which is evenly divided into virtual grids. Each box or cell includes a cluster of sensor nodes, and a main node of the cluster is chosen among them as it is done in [32]. The header node is responsible for receiving data from the multi-hop wireless sensor nodes, and sending them to the UAV or mobile sink node. The UAV visits each cluster in different regions, collecting data from the sensor nodes of each cluster. This avoids collecting the information from all the sensor nodes, telling the trajectories only where the main nodes of each cluster are located. The proposed model is based on calculating the *VoI* animal monitoring, used to know which clusters should visit the UAV. The definition of the trajectory is carried out by means of a Markovian decision process model (MDP), where the states represent the grids and actions that lead to transitions of deterministic state. The performance results in this article are based on the measurement of the volume of information (or *VoI*), packet loss, and packet delays, which depends on the trajectory model chosen for the UAV, but not the power consumption. The work in [42] presents an investigation where it proposes a wireless sensors and actors network (WSAN), for the analysis of the social network of the society of apes through the animal’s mobility. In their experiments, each gorilla is equipped with a wireless sensor node, while the “silverbacks” or leader gorillas are equipped with the mobile CHs as a sinks nodes.

In this proposal, the UAV CHs are equipped with the sink mobile nodes to have a non-invasive monitoring system since collecting the data directly from the “silverbacks” or any other similar animal is complicated.

Hence, in this manuscript, a mathematical model based on a discrete-time Markov chain (DTMC) is proposed to calculate the average energy consumed by the UAV and the cluster-based WSN. The drone flies in two different pre-established strategies to collect data from each cluster leader or coordinator, with previously known locations. These strategies are aimed at reducing the energy consumption either at the UAV or at the WSN. System simulation is also implemented for validation, and the energy consumption analysis is made to determine which of the two schemes used is more efficient for different conditions.

## 3. System Model

**Figure 3:**
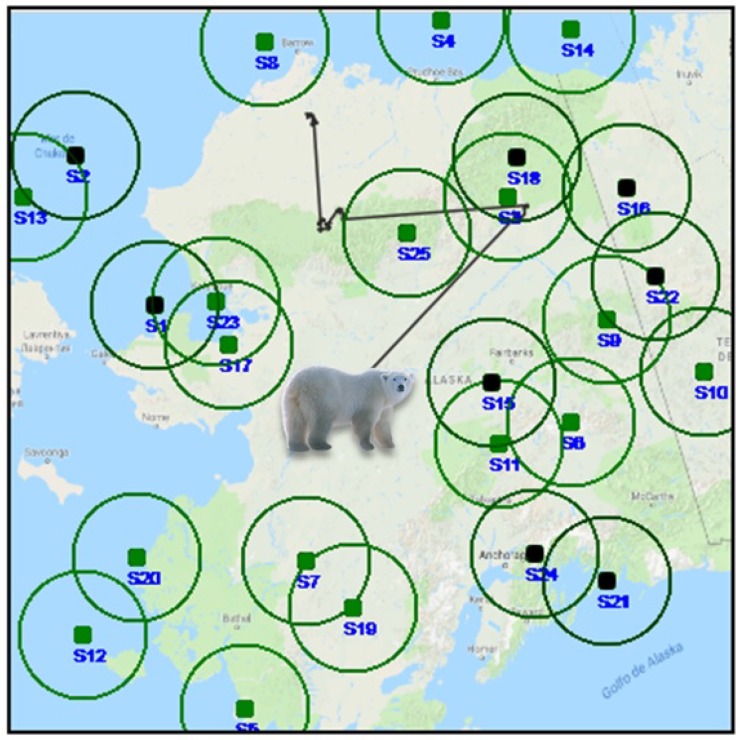
Example of a polar bear trajectory passing inside the $R_{cov}$ of the WSN inside an $A_{obs}$.

In this section, the system operation of the WSN to monitor endangered animals and the two proposed data gathering schemes through a single UAV is described in detail. Additionally, the main assumptions of this work to calculate the average energy consumption of the total system are given. To this end, it is considered a real scenario where *N* numbers of sensors are randomly deployed following a uniform distribution in a specific natural environment or habitat, where the animals live, bounded by $A_{obs}$ and the human access is difficult or even dangerous. It is supposed that these static sensors are going to maintain periodically monitoring the animal under study, detecting the sensors attached to a device that is placed on them, like collars or bells that can measure any type of vital sign, like temperature, blood pressure, or GPS coordinates or by using specific sensors that can detect these animals. It is assumed that when the animal passes and is inside of any node’s $R_{cov}$, the static sensor is going to gather the stored animal information as depicted in Figure 3.

This work proposes that the WSN data transfer method between CMs and CHs be based on clustering with star topology, and the data transfer method between CHs and the airmobile sink node (recollecting UAV) is proposed to be based on the WSN-O or UAV-O schemes divided into two main stages: Cluster formation (CF) and steady-state (SS) stage. For both schemes, the WSN forms the number of clusters ($N_{ch}$), each with a CH, previously chosen depending on the needs of the system and with many CMs ($N_{cm}$). In the CF stage, it is proposed that all the sensors can receive and transmit the packets, with a high energy consumption due to the large area of observation, to all the other nodes in the WSN using the slotted aloha protocol (S-ALOHA), so, the first $N_{ch}$ nodes that transmit a successful packet becomes the CHs. Then a CH coverage radio is established to choose the sensors inside each CH as the respective CMs. Once the CF stage is complete, then the SS process is developed and here the CMs can now transmit their data to their respective CH, gathered through the UAV with the two schemes mentioned above.

The WSN-O scheme, shown in Figure 1 proposes that every CH is going to recollect the packets from its respective CMs using the time division multiple access (TDMA) protocol during a round, which is the minimum time (*R*) for that, all the CMs of every CH, can transmit at least one packet, so there are going to be a random *F* number of frames (number of times all CMs transmit one packet of each CH per round). During this stage, the CHs are receiving and buffering all the data from all the CMs and these CMs are transmitting with less power than in the CF stage to its CH. When they are not transmitting in the corresponding TDMA slot, the CMs are in sleep mode consuming much less energy. When the *R* time is finished, it is proposed that the UAV flies from a specific far away from the base station (BS) to every CH, recollect the storage data, and return to the BS. The consumption energy that the UAV has visiting all the CHs and the energy drained from the CHs to send the data to the UAV is considered too. Like in WSN-O, the UAV-O scheme, depicted in Figure 2 uses the TDMA so that the CMs transmit their packet to their respective CHs, but here, it is randomly selected so that one main CH or CH_sink_ receives all the data from all the CHs every time a frame is transmitted. Hence, the CHs in this stage is going to spend more energy than in the WSN-O and the CH_sink_ needs to have a bigger buffer. Then, once the round is complete, it is proposed that the UAV flies from its BS to only CH_sink_, recollect all the storage data and return to its BS.

So two main stages are established for this process:

1 Cluster formation (CF), where each node in the network assumes its role as either CM or CH;
2 Steady state (SS), where CMs send their packets to their CHs, and where CHs send their gathered data to the sink UAV.

In the CF stage, nodes become either CHs or CMs. This is done using the S-ALOHA as follows: At the beginning of the CF phase, all nodes transmit a control packet (only the node’s ID is required at this stage at the beginning of each time slot of duration $P_{slot}$ seconds with probability τ).

Once a node successfully transmits, i.e., only one transmission occurs in a given time slot, its packet is correctly received and that node does not transmit any more packets for the remaining of the CF procedure. If there are multiple transmissions in a time slot, all packets involved in the collision have to re-transmit in a future time slot. The CF phase ends when all nodes have successfully transmitted their packet. As such, all nodes are aware of the rest of the nodes and, since all nodes received the control packets of the rest of the nodes, they can estimate their distance to each other.

**Figure 4:**
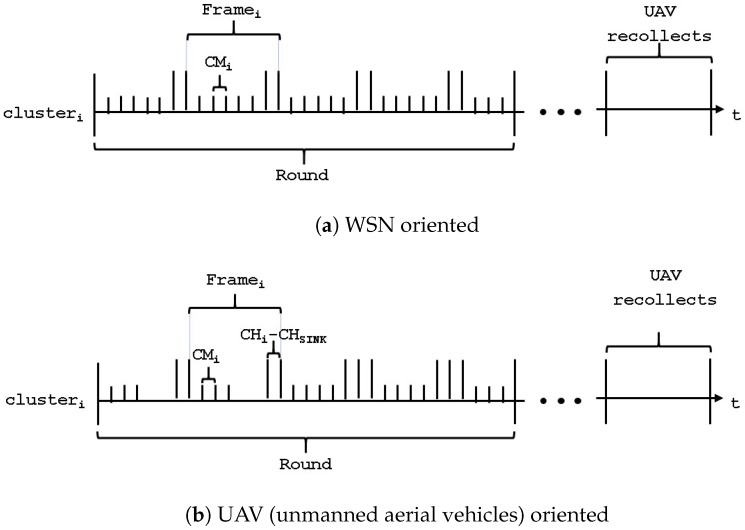
Slot times, packets, frames, and round structure in the SS (steady-state).

Additionally, the first $N_{CH}$ nodes that successfully transmit their control packet become CHs and the rest take the CM role by associating themselves to the closest CH. Note that the energy consumed by the nodes to transmit this control packet, $E_{Tx}^{CF}$ energy units, is proportional to the size of the monitored area, $A_{obs}$ and the packet size, which in this case is rather small. When nodes that have not successfully conveyed their control packet do not transmit, with probability 1−τ, or when they have already transmitted their packet and are waiting for the transmission of the rest of the nodes, they consume less energy, $E_{Rx}^{CF}$ energy units, since they are in reception mode. A round is defined as the time it takes to finish the SS stage and a frame is defined as the time it takes for all CMs within a cluster to send their information to their CH. Figure 4 shows the structure of the time slots, packets, frames, and round for the WSN-O and UAV-O schemes in the SS stage.

The transfer of the data payload to the CHs from the CMs process is carried out in the SS stage as well as the data gathering process from the CHs to the sink UAV but in two different ways depending on the two proposed schemes. The UAV is considered to be a quadrotor because of its physical characteristics and the type of flight that this aircraft has, fulfilling the needs of the proposed recollecting method. The energy consumption of the drone is analyzed and computed using an analytical model based on the backstepping method in the UAV energy consumption model section.

### 3.1. WSN Oriented

The TDMA technique is used in the SS phase of this scheme so that all the CMs can transmit all the information regarding the monitored animal during a slot time $P_{slot}$, defined by the bitrate and packet size of the selected transceiver device, to their respective CHs. In this scheme, the transmitting CMs consume $E_{Tx}^{WSN-O}$ energy units, which is less than in the CF phase (because of the smaller area of the cluster). Additionally, unlike the CF phase, CMs that are not transmitting are in sleep mode, consuming $E_{sleep}^{SS}$ energy units. Meanwhile, the CHs are receiving and storing the data coming from their respective CHs and consuming $E_{Rx}^{WSN-O}$ energy units. When one frame is completed (all CMs send packets to their respective CH), the CHs occupy one time slot to acknowledge to all CMs the received data and then all the CMs can transmit again their next packets until, the round time *R* expires. At this point, all clusters have to be formed again following the CF procedure detailed above. Once the round is concluded, as described above, one UAV recollects the stored data as follows:

- The UAV starts his flight, from the BS to all CHs, when round time *R* expires;
- The UAV has a previously-defined path flight, which entails a route calculated offline so that the UAV can visit all the CHs in the WSN in only one flight;
- Then, when the UAV is static and above each CH, it gathers the stored information from them, spending some flight time and;
- Once the UAV has visited all the CHs and gathered the data from them, it returns to its BS.

**Figure 5:**
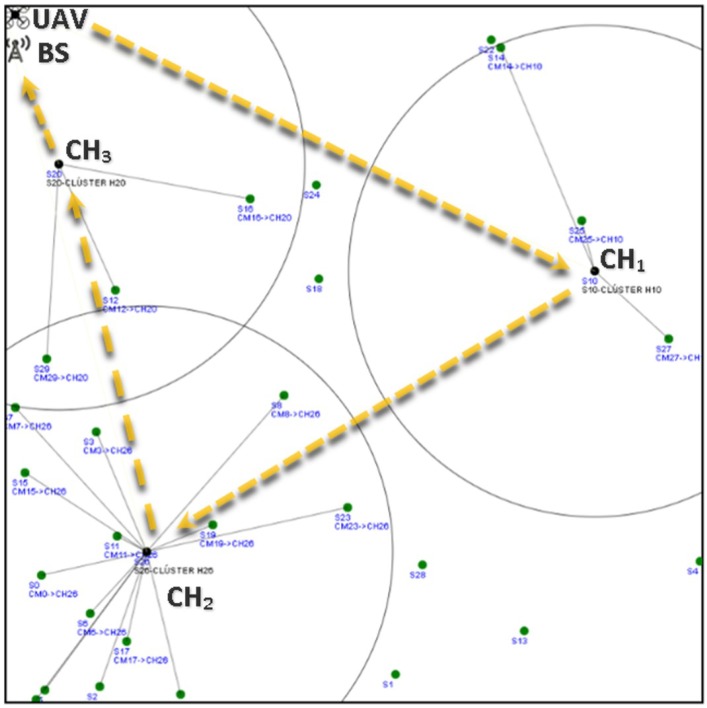
Recollecting data from the WSN example for N=30 and $N_{CH}=3$, in the SS for the WSN-O.

Figure 5 shows an example of the UAV path above the WSN for this scheme. It is important to note that, unlike conventional clustered based WSNs, CHs do not transmit the recollected data to the sink. By avoiding this, we aim at reducing energy in the sensor network.

### 3.2. UAV Oriented

In this data collection scheme, the TDMA protocol is used to send data from CMs to their respective CHs, but unlike the WSN-O scheme, when a frame is complete, each CH sends the data recollected in the frame, to one CH_sink_. This CH_sink_ is previously and randomly selected from all the CHs in the WSN. So, during the entire round time, *R*, the CH_sink_ is storing all the data frames from all the other CHs, including its own, so, the CHs are spending more energy than in the WSN-O case, because they are sending data periodically at the end of every frame and consuming $E_{Tx}^{UAV-O}$ energy units to reach the CH_sink_ throughout the observation area and this CH_sink_ is consuming $E_{Rx}^{UAV-O}$ energy units each frame. Once the round is expired, the data gather method follows the next steps:

- Quadrotor launches from the BS;
- The UAV’s path is designed to visit directly only the CH_sink_;
- The UAV recollects the information from this CH, spending more time above the node, than in the WSN-O method, because of the larger size of data stored;
- Once finished the recollection, the UAV returns to its BS.

**Figure 6:**
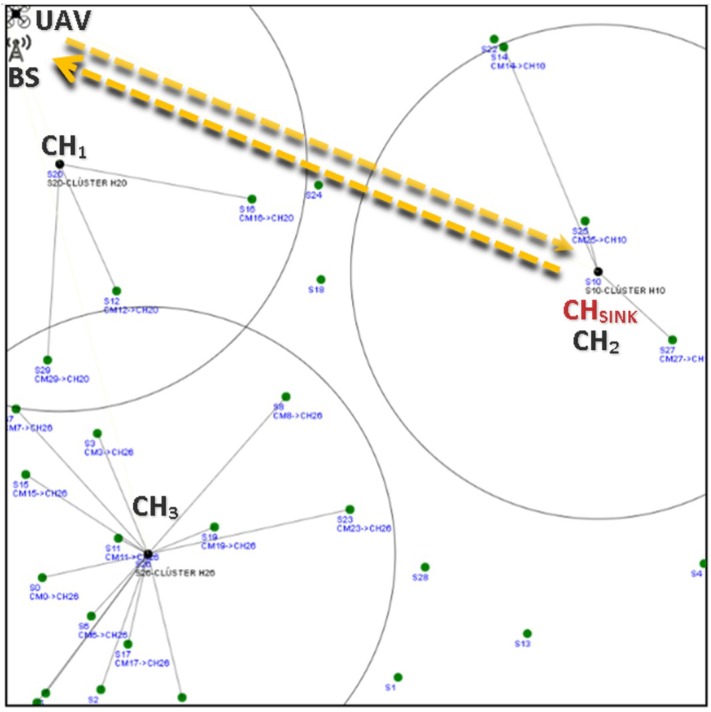
Recollecting data from the WSN example for N=30 and $N_{CH}=3$, in the SS for the UAV-O (UAV-oriented).

Figure 6 shows an example of the UAV path above the WSN for the described scheme. Also note that, unlike conventional clustered based WSNs, in this scheme, the energy consumption is lower since not all CHs transmit the data to the sink node. Instead, one CH waits for the mobile sink (drone) to arrive, making a much shorter transmission. Hence, we believe that in both data collection schemes, an energy consumption reduction is achieved employing the use of UAVs.

### 3.3. Energy Consumption Model

In order to calculate the average system consumption energy, the model provided in [43] is used, where the energy for the transmission, reception, and sleep mode activities are defined based on the observation area, $A_{obs}$, and packet size. Then, to simplify the mathematical model, these real energy values are normalized to obtain energy units (EUs) quantities. The sensor nodes selected for the WSN are based on the 802.15.4 or Zigbee standard because it is one of the most used and suitable for the animal monitoring through WSN application [44,45], due to, among others characteristics, its coverage radio, bitrate, simplicity for CF configuration, weight, size, and energy used. Hence, the xBee Pro 2sc 2.4Ghz model is chosen from the Digi company and in Table 1 we show the main specifications.

Building on this, if it is assumed that in the CF stage the nodes need to transmit in boost mode (45 mA) to reach all the other nodes in the entire monitored area and the energy consumed by the nodes is 10 EUs. Then, it is easy to calculate with a simple relation the consumed energy by other activities of the sensors as:

- Transmitting consumption energy in CF stage is $E_{Tx}^{CF}=10$ EUs;
- Receiving consumption energy in CF stage is $E_{RTx}^{CF}=7$ EUs;
- Transmitting consumption energy in SS stage is $E_{Tx}^{CF}=8$ EUs;
- Receiving consumption energy in SS stage is $E_{Rx}^{CF}=6$ EUs;
- Sleep consumption energy in CF and SS stages is $E_{Sleep}^{CF}=E_{Sleep}^{SS}=1\times 10^{-3}$ EUs.

## 4. UAV Design

While the quadrotor is on flight, it drains the battery energy ($E_{UAV}^{SS}$) and this has been characterized by the proposed model in this section considering, among others parameters, the capacity and time of discharge of the battery, size, and weight of the UAV, as well as the vertical and horizontal speed, altitude, distance, and flight path. In the model, the backstepping control approach is used and the actuators are supposed to be DC motors instead of brushless motors for the UAV. DC electric motors were used for reasons of simplicity and practicality [46] and though the energy consumption is not efficient, with the ideal parameters it is suitable for the task.

The quadrotor dynamics is approximated with an approximate linearization. A standard linear controller is designed because it does not need the condition of well-known model parameters to balance the nonlinearities, like atmospheric turbulence or system degradation [47]. The backstepping technique is used to relieve the disadvantages of the feedback linearization method and simplifies the control task, avoiding very complex systems when doing direct input-output control [48]. The UAV as a quadrotor is an underactuated system, that has six degrees of freedom (x,y,z,θ,ϕ,ψ) and four actuators. The quadrotor is assumed as a rigid body.

**Figure 7:**
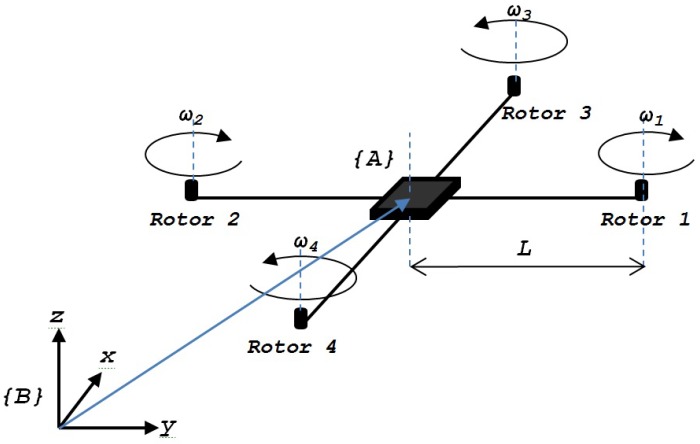
Quadrotor configuration of the body axis system.

Figure 7 shows a schematic diagram of the quadrotor with the inertial reference system {A} and the reference system {B} attached to the mass center of the quadrotor body [49].

Let us define *m* as the total quadrotor mass, *L* as the total distance from the center of mass of each rotor, $r={\left[x,\;y,\;z\right]}^{⊺}$ as the position vector, $I\in R^{3\times 3}$ as the inertial moments of quadrotor with respect to {B}, and ω∈R as the angular velocity in the inertial frame [50].

**Figure 8:**
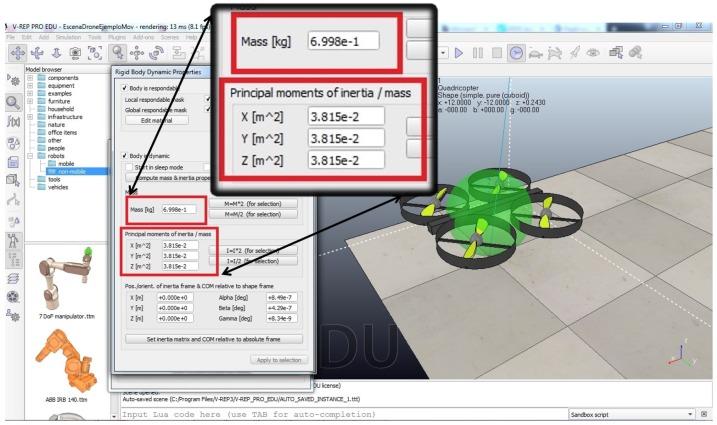
Principal moments of inertia of quadrotor obtained in *Vrep* software.

Then, from Equations (1) and (2), the trajectory model and DC motors dynamic model are provided. ϕ, θ, and ψ represent the pitch, yaw, and roll rotational movement of the quadrotor respectively while *z* is the vertical position; *z* is only defined because it is assumed, for simplicity, that the left and right direction of the UAV can be determined by the rotational angles instead of *x* and *y*:

(1) $$\left[\begin{array}{c} \ddot{\phi }\\ \ddot{\theta }\\ \ddot{\psi }\\ z \end{array}\right]=\left[\begin{array}{cccc} J_{1} & 0 & 0 & 0\\ 0 & J_{2} & 0 & 0\\ 0 & 0 & J_{3} & 0\\ 0 & 0 & 0 & J_{4} \end{array}\right]\left[\begin{array}{c} U_{1}\\ U_{2}\\ U_{3}\\ U_{4} \end{array}\right]$$

(2) $$\left[\begin{array}{c} U_{1}^{*}\\ U_{2}^{*}\\ U_{3}^{*}\\ U_{4}^{*} \end{array}\right]=\left[\begin{array}{cccc} k_{F} & k_{F} & k_{F} & k_{F}\\ 0 & L & 0 & -L\\ -L & 0 & L & 0\\ k_{M} & -k_{M} & k_{M} & -k_{M} \end{array}\right]\left[\begin{array}{c} \omega_{1}\\ \omega_{2}\\ \omega_{3}\\ \omega_{4} \end{array}\right]$$

where $U_{i}$ are the control inputs, $U_{i}^{*}$ are the feedforward control inputs, $J_{i}$ the inertia moments, $K_{F}$ and $K_{M}$ the thrust and rotation coefficients respectively, and *L* the lever arm longitude. Then, a set of proportional derivative (PD) controls for the trajectory are described as:

(3) $$\begin{array}{cc} U_{1} & =\frac{1}{J_{1}}\left[{\ddot{\phi }}^{*}-k_{d_{1}}\left(\dot{\phi }-{\dot{\phi }}^{*}\right)-k_{p_{1}}\left(\phi -\phi^{*}\right)\right]\\ U_{2} & =\frac{1}{J_{2}}\left[{\ddot{\theta }}^{*}-k_{d_{1}}\left(\dot{\theta }-{\dot{\theta }}^{*}\right)-k_{p_{1}}\left(\theta -\theta^{*}\right)\right]\\ U_{3} & =\frac{1}{J_{3}}\left[{\ddot{\psi }}^{*}-k_{d_{1}}\left(\dot{\psi }-{\dot{\psi }}^{*}\right)-k_{p_{1}}\left(\psi -\psi^{*}\right)\right]\\ U_{4} & =\frac{1}{J_{4}}\left[{\ddot{z}}^{*}-k_{d_{1}}\left(\dot{z}-{\dot{z}}^{*}\right)-k_{p_{1}}\left(z-z^{*}\right)\right] \end{array}$$

where $\phi^{*}$, $\theta^{*}$, $\psi^{*}$, and $z^{*}$ are the desired position of the quadrotor and a proportional integral (PI) for the DC motor control is described by:

(4) $$\dot{\omega_{i}}=-\frac{b_{i}}{I_{i}}\omega_{i}+\frac{V_{i}}{I_{i}}-\frac{d_{i}}{I_{i}}$$

where i=1,⋯,4 is the number of the motor, $I_{i}$ is the current, and the $V_{i}$ is the force electromotive (FEM) induced to each motor, $b_{i}$ friction coefficients, $d_{i}$ disturbances, and $\omega_{i}$ the angular velocity of each motor. Hence, with the backstepping method, the angular velocity of each motor is calculated according to the UAV’s movement and proposed trajectory, which is calculated by the two control stages described above. Building on this, the energy consumed by each rotor is calculated using Ohm’s law knowing the induced voltage and current to this DC motors. A simulation in Matlab Simulink (vR2015a) software was proposed to calculate the energy consumed by the four motors of the UAV using the described model. The electrical and physical UAV parameters used are shown in Table 2. The main inertia moments $I_{x}$, $I_{y}$, and $I_{z}$ were calculated using a physical model of the quadrotor selected on the Vrep (PRO EDU v3.5.0) software as shown in Figure 8. Vrep was used to simulate some of the desired trajectories of the UAV (considering, among others, size, weight, materials, distance, velocity, Bézier trajectories [51], and time flight) until it had the best possible flight stabilization.

**Figure 9:**
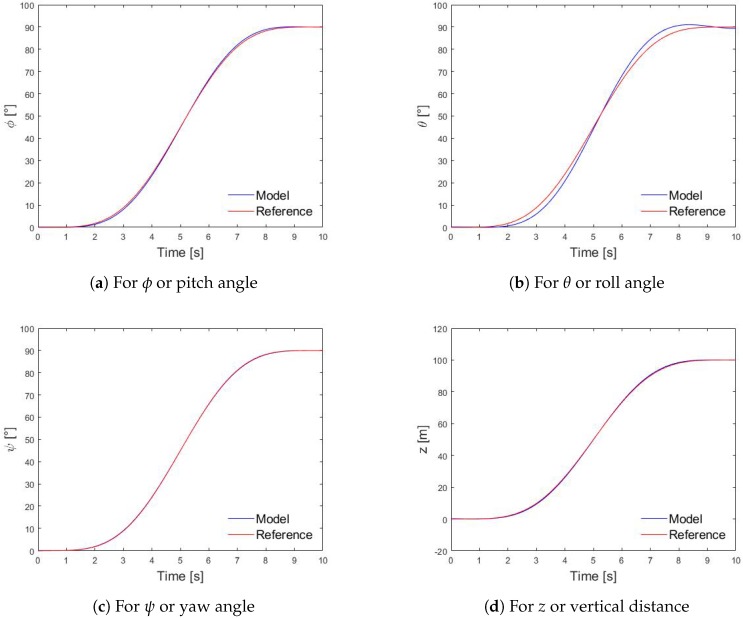
Model and desired trajectories tracking results.

Then the quadrotor control model scheme with Bézier trajectories is depicted in Figure 9 which shows that the calculated ϕ, θ, ψ, and *z* coordinates are approximately equal to the reference coordinates or the desired ones which they were calculated with the Bezier trajectories equations [52] and using, as an example, an observation time of 10 s, $\phi^{*}=90{}^{\circ}$, $\theta^{*}=90{}^{\circ}$, $\psi^{*}=90{}^{\circ}$, and $z^{*}=100$ m. As such, it is concluded that this control method to define the trajectories is valid.

### Energy Consumption Model

**Figure 10:**
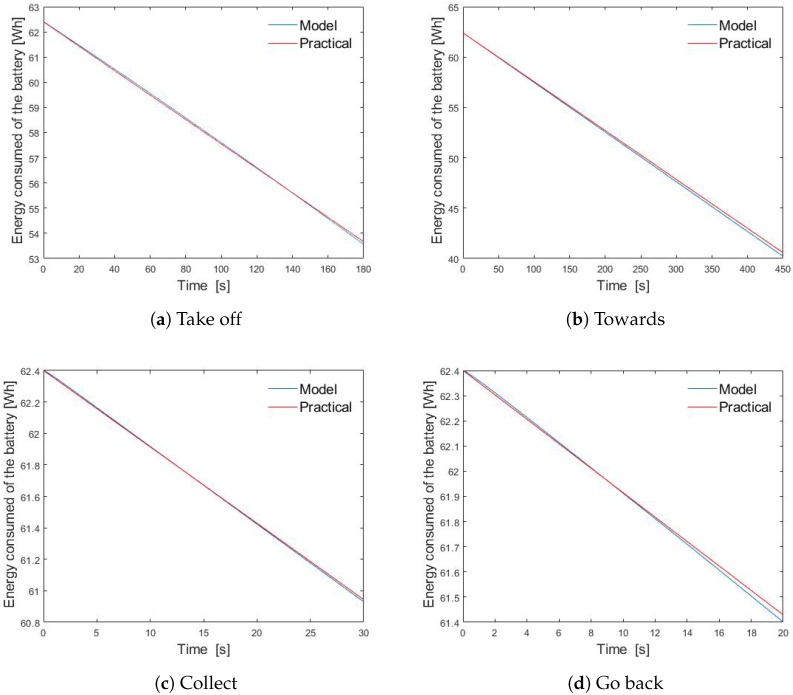
Consumed energy for each trajectory.

Now, the proposed UAV’s energy consumption model is validated. To this end, it is compared to the energy values reported in practical tests, like in [53], where only the battery characteristics and the time flight are considered which is calculated as:

(5) $$P_{prac}\left(t\right)=V_{batt}\;\cdot \;\left(\frac{\left(I_{batt}\;\cdot \;time_{obs}\right)}{time_{total}}\right)$$

where $P_{prac}\left(t\right)$ is the consumed power drained from the battery, $V_{batt}$ is the battery voltage, $I_{batt}$ is the battery current, $time_{obs}$ is the observation time, and $time_{total}$ is the total time in which the battery drains out, according to its respective datasheet. The energy consumed for the analytical model and practical case was calculated assuming that the UAV has a Lipo battery of 5200 mAh, 12 V, 60 Wh with a discharge rate of approximately 3-c (20 m or 1200 s) and vertical and horizontal speed of 5 m/s and 8 m/s respectively. Hence, a flight path from point A to point B was established simulating the quadrotor process and energy consumption calculated from the proposed model and with the simple and common model from (5). The process and energy results are presented as follows:

1 Take off in 180 s (vertical flight z= 0–250 m, ϕ= 0) Figure 10a;
2 Moving towards 3.6 km in 450 s (horizontal flight z=10 m, ϕ= 0–90) Figure 10b;
3 Keep static in the air in 30 s (recollecting z=250 m, ϕ= 0) Figure 10c;
4 Rotate in 20 s (z=250 m, ψ= 0–180) Figure 10d;
5 Go back (same as 2);
6 Landing in 180 s (vertical flight z= 250–0 m, ϕ=0 (same as 1)).

The sum of the time intervals and energy drained in each trajectory results in the total energy consumed in a specific period. In practical terms and, based on Equation (5), this UAV will discharge its Lipo battery of (60 Wh) in 20 min and the model presented here will discharge in approximately 1040 s or 17.33 min. Then, the proposed consumption energy model is to be validated. From Figure 10 it is shown that for the recollecting mode, the drone consumes about 1.5 W in 30 s, for the horizontal or towards flight it consumes 20 Wh in 450 s and for the launching/landing mode it consumes approximately 8 Wh in 180 s. Hence for the defined time slot $P_{slot}=4mS$, the horizontal flight is going to consume about $1.77\times 10^{-3}$ Wh per slot, and if this is considered the highest power consumption, it can be established that $1.77\times 10^{-3}$ Wh is equivalent to 10EUs. By normalizing the horizontal flight consumed energy, the other energy units can be defined as:

- The consumed energy for the recollecting mode is $E_{gather}^{UAV}=1.13\frac{EUs}{slot}$;
- The consumed energy for the vertical flight mode is $E_{vertical}^{UAV}=1\frac{EUs}{slot}$;
- The consumed energy for the horizontal flight mode is $E_{horizontal}^{UAV}=10\frac{EUs}{slot}$.

## 5. Simulation Model

After the UAV consumption model was characterized, an in-house, non-commercial simulator (programmed in C++ language) is developed to consider the data collection from the WSN to the UAV considering the two recollecting schemes proposed in this work, based on the system described in Section 3.

The pseudo-code presented in Algorithm 1 shows the general procedure of the system considering all the system dynamics of packet transmissions, packet receptions, sleep times, and so on. The deploySensors() function generates random positions for each node inside the $A_{obs}$ following a uniform distribution and each of them has a $R_{cov}$. Based on this simulation, the total energy consumption is calculated by the WSN in the CF stage by the consumedEnergyCF() function, which sums the energy consumed by nodes when they transmit, sleep, or idle respectively and the initial variables and parameters given by the specific application, i.e., the number of nodes (*N*), the number of CHs ($N_{CHs}$), the coverage radius of the CHs, or the size of the observation area ($A_{obs}$).
**Algorithm 1** Simulation algorithm for CF process1:Initialize $R_{cov}$, $N_{ch}$, *N*, L= 100,000, consumedEnergyCF(), collisionCount, trOne=0, numSuccessSlots=0, $A_{obs}$;2:*deploySensors()*, deploys *N* sensors with uniform random distribution;3:**for**l=0 to l<L **do**4: Define k=0, σ=0.1, τ=0.1, successfulNode=0, $N=N_{new}$, $N_{col}$,nodoId=[], nodoCH;5: Program event objectType[0,1]=“slot”;6: **if** $successfulNode<N_{new}$ **then**
7:  Delete first event objectType.splice[0];8:  **if** objectType[0]==slot" **then**9:   Program next event objectType.push[0];10:   tr=0, trOne=0;11:   **for** j=0 to $j<N_{new}$ **do**12:    $u=random\left(1e^{6}\right)/1e^{6}$
13:    **if** u<σ **then**
14:     tr++
15:    **end if**
16:   **end for**
17:   **for** i=0 to tr **do**18:    Choose 1 from *N*: nodoId[i]=random(0,N)19:    **for** j=0 to successfulNode[j] **do**20:     **if** nodoId[i]==successfulNode[j] **then**
21:      repeat if succesfulNode[i] is already successful: i=i−122:     **end if**
23:    **end for**
24:    consumedEnergyCF();25:   **end for**
26:   **if** tr==1 **then**
27:    trOne=1;28:   **end if**
29:   collisionCount=0;30:   **for** j=0 to $j<N_{col}$ **do**31:    $u=random\left(1e^{6}\right)/1e^{6}$
32:    **if** u<τ **then**
33:     tr++, collisionCount++;34:    **end if**
35:   **end for**
36:   **if** tr==1 **then**
37:    numSuccessSlots++;38:    $N_{col}=N_{col}-collisionCount$;39:    $N_{new}=N_{new}+collisionCount$;40:    successfulNode[i]=nodoId[0];41:    k=k+1;42:   **end if**43:   **if** tr>=2 **then**
44:    $N_{col}=N_{col}+trOne$;45:    $N_{new}=N_{new}-trOne$;46:   **end if**
47:   **if** $succesfullNode\left[i\right].length>=N_{n}$ **then**
48:    k=0;49:   **end if**
50:   **for** j=0 to $j<N_{ch}$ **do**51:    k=0;52:    nodoCH[j]=successfulNode[j];53:    and the rest are CMs54:   **end for**
55:  **end if**
56: **end if**
57:**end for**

After the CF phase is complete, the SS stage is coded in Algorithm 2. Here the *transmitTDMA()* function calculates the energy consumed by the CMs while they are sending their packets to their respective CH but the method used to collect this data is different depending on the selected scheme.
**Algorithm 2** Simulation algorithm for SS process1:Initialize scheme, recollectingTime, *R*, slotTime, totalSlotTDMA=fracRP, ranuraTDMA=0, $N_{ch}$, *N*, timeVertUAV, $totalSlotTDMA=\frac{UAValtitude}{speedVertUAV}$, visitCHs=false, visitCHuavo=false, L=100,000, consumed energies in SS;2:**for**l=0 to l<L **do**3: **if** scheme==“WSN−O” **then**
4:  **if** slotTDMA<totalSlotTDMA **then**
5:   transmitTDMAwsno();6:   energyConsumedTDMAwsno();7:   slotTDMA++;8:  **end if**
9:  **if** timeVertUAV<tiempoVertTotalUAV **then**
10:   vertFlightUAVwsno();11:   energyConsumedVertUAVwsno();12:  **end if**
13:  **if** visitCHwsno==false **then**
14:   horFlightUAVwsno();15:   energyConsumedHorUAVwsno();16:   **if** visited CHs by UAV == $N_{CH}$ **then**17:    visitCHwsno=TRUE
18:    return UAV to BS;19:   **end if**
20:  **end if**
21: **else**
22:  **if** slotTDMA<totalSlotTDMA **then**
23:   transmitTDMAuavo();24:   energyConsumedTDMAuavo();25:   slotTDMA++;26:  **end if**
27:  **if** timeVertUAV<tiempoVertTotalUAV **then**
28:   vertFlightUAVuavo();29:   energyConsumedVertUAVuavo();30:  **end if**
31:  **if** visitCHuavo==false **then**
32:   horFlightUAVwsno();33:   energyConsumedHorUAVwsno();34:   **if** UAV visits CH_sink_ **then**35:    visitCHuavoo=TRUE
36:    return UAV to BS;37:   **end if**
38:  **end if**
39: **end if**
40:**end for**

The UAV parameters considered in the simulation are: The vertical and horizontal speed of the UAV, flight altitude, quadrotor energy consumption, the slot time, packet size, bit rate, and nodes energy consumption, among others.

Once the round is completed, the UAV launches and takes the vertical flight until it reaches the desire selected altitude (altUAV). Then the UAV follows the path flight defined by the horFlightUAV() function, which is also modified, depending which scheme was selected and then, the quadrotor returns to the BS.

Now, the data collection by the UAV considered in the simulations is described in detail.

- **WSN oriented function**. In this scheme, the transmitTDMAwsno() function simulates the packets being transmitted by the CMs to their respective CH employing the TDMA protocol with time slots of duration $P_{slot}$ and the energyConsumedTDMAwsno() function calculates the consumed energy in this process. Thus when all the members of each CH send their packets, a TDMA slot is occupied by the respective CH to acknowledge to all their CMs and then the process starts again until the round time *R* expires. In this process, the energy consumed by the CMs and CHs inside each formed cluster is calculated as described in the System Model section. Then, the vertFlightUAVwsno() function is used to allow the UAV to fly vertically until it reaches the given altitude and the horFlightUAVwsno() function simulates the horizontal flight. When it reaches the given altitude the horFlightUAVwsno() function simulates the recollecting data process so that the UAV visits each CH of the WSN, spending recollectingTime seconds above each sensor and the energyConsumedHorUAVwsno() function calculates the energy spent in this process. The energy consumed by the quadrotor and nodes in this stage is calculated as detailed before;
- **UAV oriented function**. The transmitTDMAuavo() function implements the UAV oriented scheme, in which, the selected CH acts as a sink. This CH_sink_ receives all the data from all the others CH in every frame and stores it. The energyConsumedTDMAuavo() function calculates the consumed energy in this scheme. When the round expires, the vertFlightUAVuavo() function simulates the vertical flight and horFlightUAVuavo() makes the UAV to fly directly to the CH_sink_ and recollect all the data stored, while energyConsumedHorUAVuavo() calculates the consumed energy in this process.

Furthermore, the two energies consumed in the system, calculated from the CF and SS phases are added to obtain the total average energy consumed. This simulation is performed over L= 100,000 times to calculate the total average consumed energy of the system.

Finally, the buffer size per node was calculated in terms of the number of packets (NoP), according to the selected scheme. For the CF phase, in both schemes, the NoP is equal to the total *N* nodes since only one successful packet for each node is transmitted. For the SS phase (based on the TDMA protocol), in the WSN-O scheme, the NoP is calculated by cluster, adding the packets transmitted by each CM to their respective CH, counting an extra CH time slot by frame to store their data. In the UAV-O scheme, the total buffer size is the size that the CH_sink_ needs, so the NoP transmitted by each CM is calculated to their respective CH, but uses two extra time slots by frame: One for their data and the other for the transmission to the CH_sink_, but this one is not counted as a data packet. The NoP is calculated in the Algorithm 2 with transmitTDMAwsno() and transmitTDMAuavo() functions respectively.

## 6. Mathematical Model

In this section, the mathematical model to obtain the average energy consumption in the monitoring system is described in detail. In the literature, animal tracking using WSNs has been previously modeled considering the complete network by finding the energy consumption of a group of nodes in a clustered architecture [54,55]. In this work, a similar procedure is taken. However, the advantages of using UAVs to collect data are considered. In this regard, the use of a drone can be easily justified in large observation areas, where CHs have to perform highly costly transmissions to convey their gathered information to the sink. As such, the derived model can be used to select either the WSN oriented scheme (when the energy required from the nodes to reach the sink is very high) or the UAV oriented (when the energy consumed by the flight of the drone is very high).

**Figure 11:**
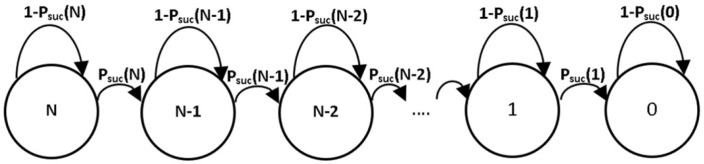
Markov chain of the mathematical model for CF (cluster formation).

In the proposed schemes (WSN-O and UAV-O), the CF procedure is the same. Indeed, up to this point where members transmit their data to their respective head, the drone is not involved. Then, the mathematical model proposed to calculate the energy consumption in the CF phase is described by a DTMC transitory Markov chain as shown in Figure 11, where *N* is the total number of nodes in the system and $P_{suc}\left(i\right)$ is the success probability, i.e., the probability that a single packet is transmitted in a given slot when there are *i* active nodes (nodes that have not successfully transmitted in previous slots).

Building on this, $P_{suc}\left(i\right)$ can be calculated as:

(6) Psuc(i)=i1τ(1−τ)i−1=iτ(1−τ)i−1,1≤i≤N,

where τ is the probability that a node transmits a packet. Hence, the average time that the system remains in state *i* is simply $1/P_{suc}\left(i\right)$. Then, the average absorption time of the chain, from state *N* (where all the nodes have to send their control packet) until state 0 (where all nodes have successfully transmitted their packet), ${\bar{D}}_{CF}$), can be calculated as:

(7) $${\bar{D}}_{CF}=\sum_{i=1}^{N}\frac{1}{P_{suc}\left(i\right)}=\sum_{i=1}^{N}\frac{1}{i\tau {\left(1-\tau \right)}^{i-1}}.$$

Note that during this time the system does not generate information since nodes are actively transmitting or receiving packets in each time slot. Additionally, as described in Section 3, the first $N_{ch}$ nodes that transmit successfully become CHs, and the rest become CMs, associating to the closest CH.

The average energy consumption in the CF phase is now derived. To this end, consider the two possible cases that can occur in a given time slot when *i* nodes are active, namely, success (a single packet is transmitted) or failure (multiple packets are transmitted or no packets are transmitted).

Then, in case of success, the average energy consumption, ${\bar{E}}_{suc}\left(i\right)$, is given by the transmission of a single packet, while the rest of the nodes remain silent, i.e., are in reception mode. This occurs with probability $P_{suc}\left(i\right)$, then:

$$\begin{array}{c} {\bar{E}}_{suc}\left(i\right)=\left[E_{Tx}^{CF}+\left(i-1\right)E_{Rx}^{CF}\right]P_{suc}\left(i\right)=\\ {}[E_{Tx}^{CF}+\left(i-1\right)E_{Rx}^{CF}]i\tau {\left(1-\tau \right)}^{i-1} \end{array}$$

$$\begin{array}{c} {\bar{E}}_{suc}\left(i\right)=\left[E_{Tx}^{CF}+\left(i-1\right)E_{Rx}^{CF}\right]P_{suc}\left(i\right)=\left[E_{Tx}^{CF}+\left(i-1\right)E_{Rx}^{CF}\right]i\tau {\left(1-\tau \right)}^{i-1} \end{array}$$

where $E_{Tx}^{CF}$ is the consumed energy by a node in transmitting mode and $E_{Rx}^{CF}$ in the receiving mode. Conversely, in case of failure, two or more packets are transmitted or no packets are transmitted. Then, the average energy consumed by a failure, ${\bar{E}}_{fail}\left(i\right)$, can be calculated as:

E¯fail(i)=ijτj(1−τ)i−jjETxCF+(i−j)ERxCF

In state *i*, there are in average $\left[\frac{1}{P_{suc}\left(i\right)}-1\right]$ failures. Then the average total energy consumed in state *i* can be expressed as:

(8) $${\bar{E}}_{total}\left(i\right)={\bar{E}}_{suc}\left(i\right)+{\bar{E}}_{fail}\left(i\right)\left[\frac{1}{P_{suc}\left(i\right)}-1\right]$$

and the average total energy consumed in the CF phase as:

(9) $${\bar{E}}_{total}^{CF}\left(i\right)=\sum_{i=1}^{N}{\bar{E}}_{total}\left(i\right)$$

In the SS phase, all CMs transmit their packets to their respective CH. To calculate the average energy consumption in this phase, let us consider the following parameters:

- The time required to transmit each packet is: $P_{slot}=\frac{packet_{size}}{bitrate}$ where $packet_{size}$ is the number of bits per packet and bitrate is transmission rate in bits per second, based on the Zigbee standard;
- The average number of members per cluster can be calculated as $N_{CM}=\left\lfloor \frac{N}{N_{CH}}-1\right\rfloor$, assuming a uniform node distribution in the network. Indeed, by considering that the first $N_{CH}$ nodes that successfully transmit their control packet become CH does not guarantee a good distribution of members per cluster. However, the use of more complex schemes assure a good distribution of cluster heads in the system. Hence, a good distribution of members per cluster would consume additional energy and resources. However, this issue falls outside the scope of this work, but for the interested reader in this topic, please refer to [56];
- The average number of packets per frame is calculated as $\frac{N}{N_{CH}}$, and each frame has a duration of *T* seconds;
- The average frame time in seconds is $T=P_{slot}\left(\frac{N}{N_{CH}}\right)$;
- There are $\left\lfloor \frac{R}{T}\right\rfloor$ frames per round, assuming that each round has a duration of *R* seconds.

In view of this, the average energy consumption per frame can be calculated as:

(10) $$\begin{array}{cc} E_{F} & =\left(\frac{N}{N_{CH}}-1\right)E_{Tx}^{SS}+\left\{\left[\left(\frac{N}{N_{CH}}-2\right)E_{sleep}^{SS}\right]\left(\frac{N}{N_{CH}}-1\right)\right\}+\left(\frac{N}{N_{CH}}-1\right)E_{Rx}^{SS} \end{array}$$

where $E_{Tx}^{SS}$, $E_{Sleep}^{SS}$, and $E_{Rx}^{SS}$ are the energy consumed by the devices in transmission, sleep, and receiving mode respectively. The first term describes that all CMs transmit packets to their CH, except the CH node. The second term describes that all the CMs that are not transmitting are in sleep mode in each slot except for the sensor and the CH times $\frac{N}{N_{CH}}-1$ slots. The third term is related to the consumption energy of the CH while it is receiving the data from each CM, except its energy.

Now, let consider the energy consumption in each proposed scheme.

### 6.1. WSN Oriented

In this scheme, the buffer size of each CH node must be $B_{size}^{WSN-0}=\left(\frac{R}{P_{slot}}\right)$ packets per round since the CHs must store all the packets that it receives from their respecting CMs in one round. At the end of the round, the drone collects these packets form each CH and only one CH is considered. Hence, the total energy consumed per round including the recollecting data process is:

(11) $$E_{R}^{W-O}=E_{F}\left\lfloor \frac{R}{T}\right\rfloor +E_{UAV}^{W-O}.$$

The first term is multiplied by the numbers of frames per round and the quadrotor must visit all the CHs per round consuming $E_{UAV}^{W-O}$ energy units as described in previous sections.

### 6.2. UAV Oriented

In this scheme, after each CH has received the data form its members, they have to transmit it to the selected CH sink. Then, the buffer size of this node has to be much larger than the buffer in the previous scheme. Specifically, the buffer in the UAV-O scheme can be expressed as:

(12) $$B_{size}^{UAV-O}=\left(N_{CH}^{avg}\right)\left(\frac{R}{P_{slot}}-N_{F}^{avg}\right)$$

where $N_{CH}^{avg}$ is the average number of CHs formed in CF phase, $\frac{R}{P_{slot}}$, as above, is the number of packets per cluster per round, and $N_{F}^{avg}$ is the number of frames per cluster or the number of time slots that the CHs used to send the data to the CH_sink_ defined by $N_{F}^{avg}=\frac{R}{T}$.

Then, the drone just visits one sink node and not all others CHs. The total energy consumed is calculated considering that the CHs must spend more energy transmitting packets to the CH_sink_.

Additionally, the UAV only flies directly to this single CH and recollects the data, spending more time above the sensor because the size data is larger and returns to the BS. Hence, the consumed energy per frame is:

$$\begin{array}{c} E_{F}^{U-O}=E_{F}+E_{Tx}^{CH_{Sink}} \end{array}$$

where $E_{Tx}^{CH_{Sink}}$ is the energy that is consumed by the single CH_sink_ and the total energy per round is:

(13) $$E_{R}^{U-O}=E_{F}^{U-O}\left\lfloor \frac{R}{T}\right\rfloor +E_{UAV}^{U-O}$$

the $E_{UAV}^{U-O}$ term is calculated as the average energy that is consumed by the UAV, flying from the BS, to the single CH_sink_ as described in previous sections.

## 7. Numerical Results

In this section, some relevant numerical results derived from the analytical model proposed above and the simulations is provided. The main performance parameter for the animal tracking system is the total energy consumed by the WSN green and the average buffer occupation of each node to measure the data capacity of the network.

As such, some results for this parameter for different coverage areas and multiple nodes are provided. Furthermore, this paper gives clear guidelines for the selection of the data collection schemes according to the monitored area based on the system’s lifetime.

**Figure 12:**
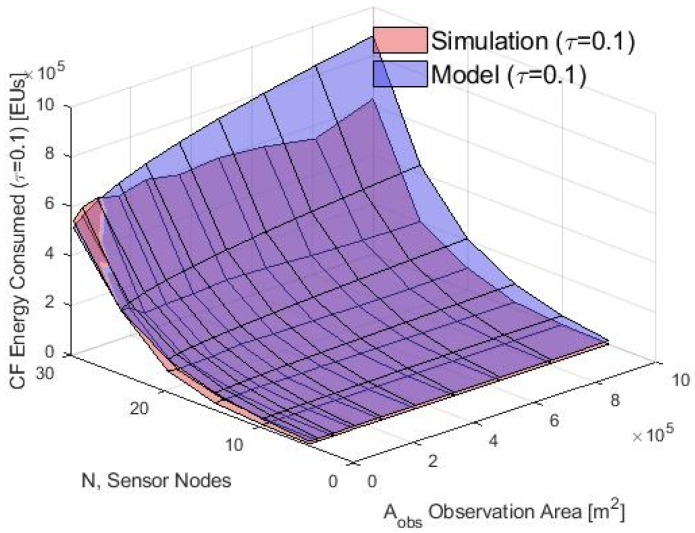
Average energy consumption in the CF phase for the simulation and model with τ=0.1.

**Figure 13:**
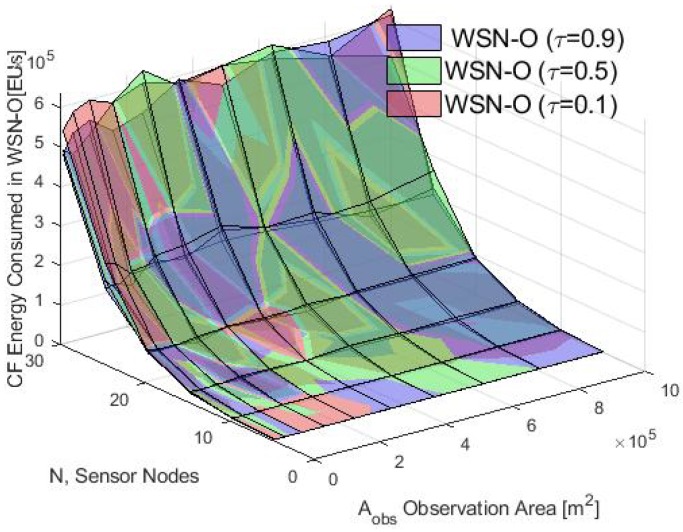
Average energy consumption with different values of τ for the simulation in the WSN-O.

**Figure 14:**
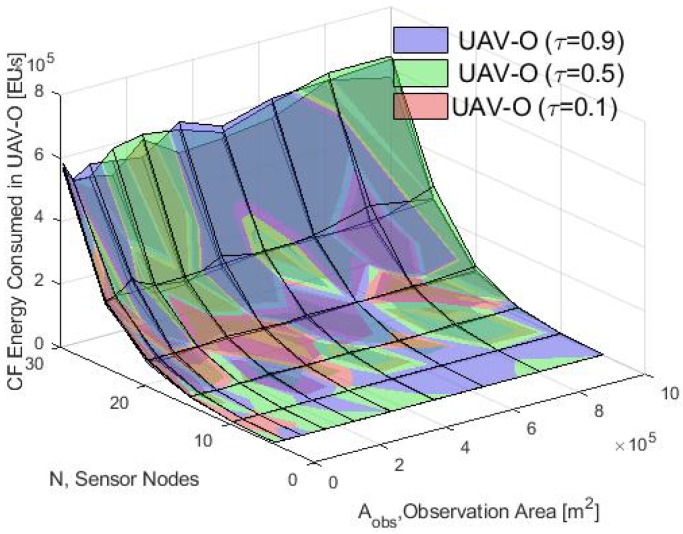
Average energy consumption with different τ for the simulation in the UAV-O.

**Figure 15:**
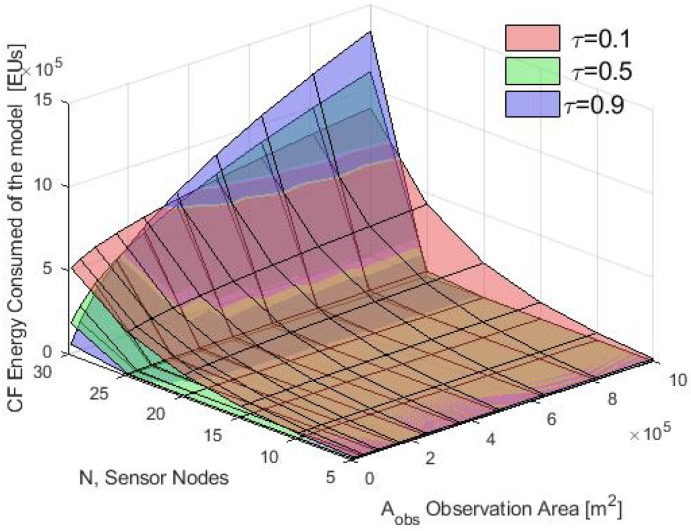
Average energy consumption with different τ for the model in the CF phase.

First, the average energy consumption in the cluster formation phase is presented. In Figure 12, the energy consumption obtained using the analytical model and the simulation results for τ=0.1 is compared. Notice that for different values of $A_{obs}$, the average of the total energy consumed by the nodes using the S-ALOHA protocol increased while the $A_{obs}$ also increased. Furthermore, when many nodes in the CF stage are deployed, the consumption energy of the WSN increases in the CF stage. Also, in Figure 13, Figure 14 and Figure 15 it is shown that if we increase the τ parameter, the energy consumed by the nodes will increase because if the probability of the packet retransmission from the nodes is increasing, the probability of collisions grows, so, the nodes spend more energy trying to retransmit those packets. Additionally, it is important to note that there is a good match between the analytical and simulation results when the $A_{obs}$ is less than $4\times 10^{5}$ m${}^{2}$ and when there are few nodes, validating the proposed mathematical model.

**Figure 16:**
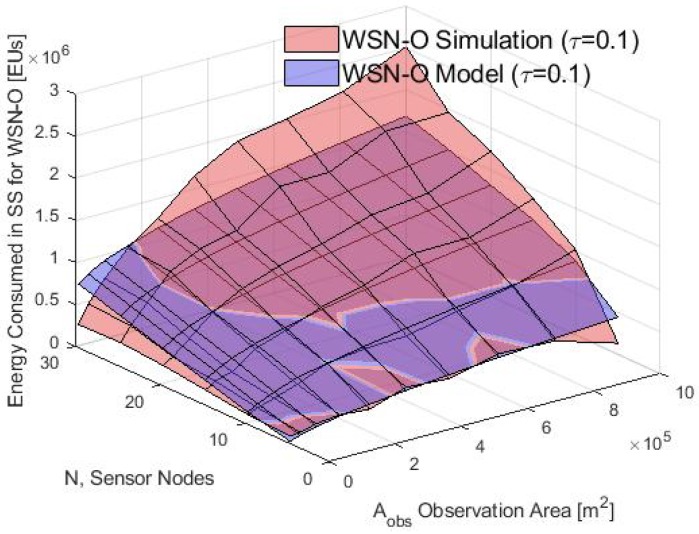
Consumed energy in the WSN-O (WSN-oriented) scheme by the model and simulation.

The system performance in the SS phase is analyzed before the UAV recollects the data. To this end, Figure 16 shows the average energy consumption calculated with the analytical model and the simulations for several values of $A_{obs}$ based on the WSN-O scheme. It can be noticed that in the simulation and the model, the energy consumption is greater when the $A_{obs}$ is increasing too because the transmitting power of the nodes is increasing while the $A_{obs}$ is larger and when there are more nodes *N*, the average number of nodes inside of each cluster increases too. It is important to note that our model is validated only for approximately less than 20 and a $A_{obs}$ less than $5\times 10^{5}$ m${}^{2}$ because the energy gap between model and simulation is growing as the parameter grows. For future work, we will review and refine the model so that this difference with the simulation will be minimal.

**Figure 17:**
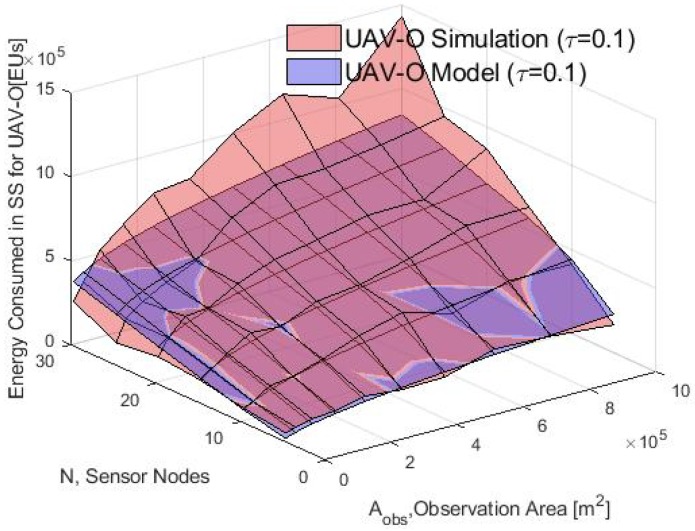
Consumed energy in the UAV-O scheme by the model and simulation.

For the UAV-O scheme, Figure 17 shows the same energy consumption behavior for different values of $A_{obs}$ and the number of nodes *N*, both for the model and for the simulation. In general, the energy consumption is lower in this scheme because once each CH store the CM’s data, they send this information to the only CH_sink_ and the rest is in sleep mode, but in the WSN-O each CH nodes that transmit their data are going to spend more energy with bigger $A_{obs}$. As above, an energy gap exists between the simulation and model for large $A_{obs}$ and more than 20 nodes occurs, hence these results are only valid for a small number of nodes and $A_{obs}$ less than $5\times 10^{5}$ m${}^{2}$ approximately.

**Figure 18:**
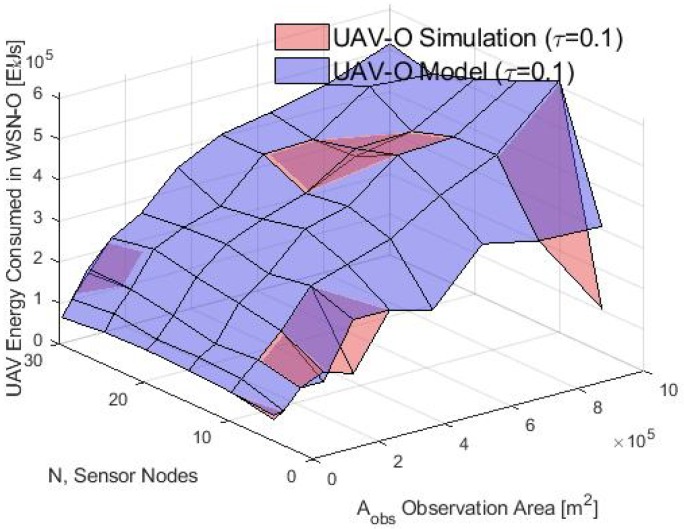
UAV energy consumption in the WSN-O scheme by the model and simulation.

**Figure 19:**
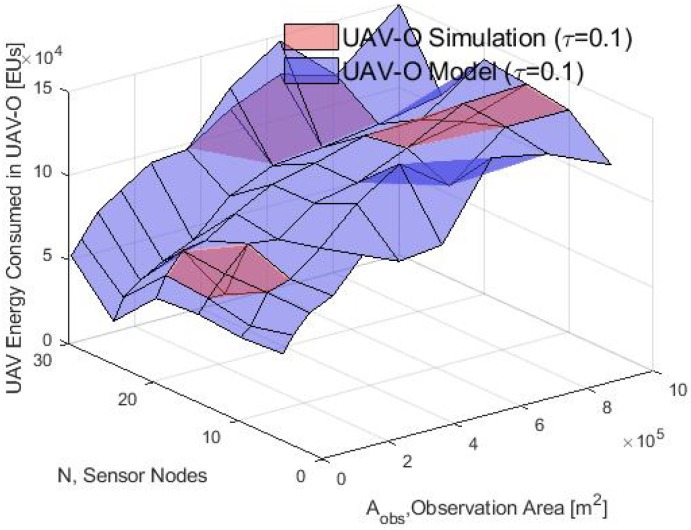
UAV energy consumption in the UAV-O scheme by the model and simulation.

Then, the UAV energy consumption simulation and model results for the WSN-O and UAV-O scheme are presented in Figure 18 and Figure 19 respectively. That for both schemes the consumption energy by the UAV increases with a bigger rate in the WSN-O scheme when $A_{obs}$ grows, because the drone has to visit all CH nodes that will become increasingly separated from each other, meanwhile in the UAV-O scheme only one needs to be visited. Furthermore, the energy drained by the UAV has increased if there are more nodes in the WSN and that it is larger in the WSN-O scheme because more nodes involve more CHs, and the UAV has to fly to more clusters. Comparing the energy consumed in the model and the simulation, we can observe that these results are valid for the thirty nodes and the entire $A_{obs}$ considered.

**Figure 20:**
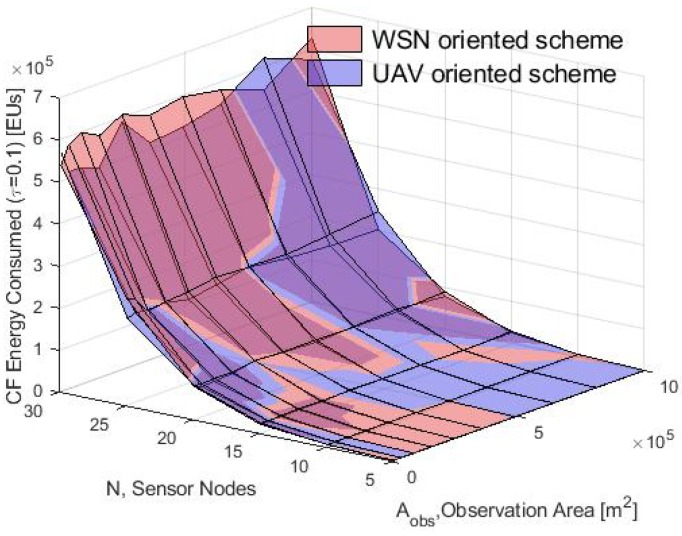
CF consumption energy for WSN-O vs UAV-O scheme in the simulation.

**Figure 21:**
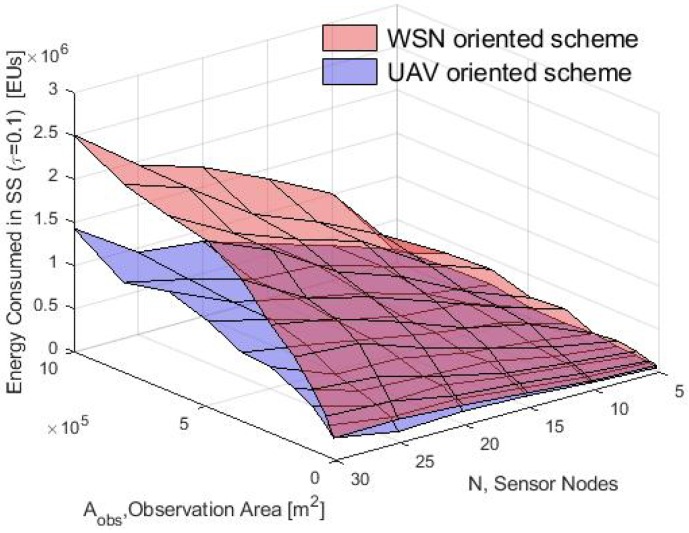
SS consumption energy for WSN-O vs UAV-O scheme in the simulation.

**Figure 22:**
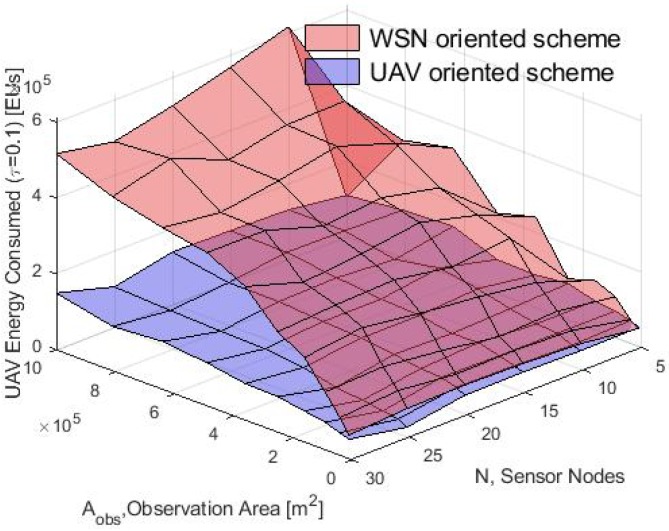
UAV consumption energy for WSN-O vs UAV-O scheme in the simulation.

In general, these results validate the proposed analytical model since there is a good match between the consumption energy obtained in the simulation for the two schemes. The performance in the simulation of both data collection schemes is compared in terms of average energy consumption in the CF, SS and UAV phases with τ=0.1 and for both the WSN-O and, the UAV-O schemes. This comparison is shown in Figure 20, Figure 21 and Figure 22. Notice that in the CF, the energy consumed by the nodes was almost the same since they used the same process to form the clusters and the energy increased when a larger number of nodes *N* was deployed and because the transmit power of the nodes was proportional to the growth of the $A_{obs}$. In the SS phase, the WSN-O scheme drained the energy nodes much faster than in UAV-O with longer observation areas and larger number of nodes. In the recollecting process, the WSN-O was also the scheme that consumed more UAV energy because of the number of clusters that had to visit all over the $A_{obs}$.

**Figure 23:**
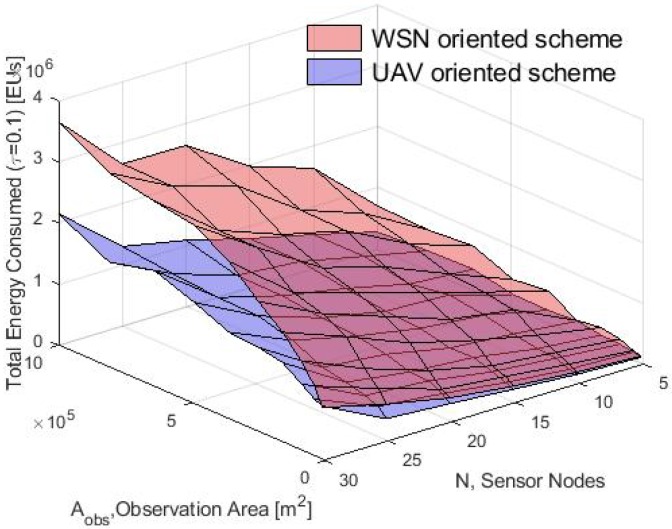
Total consumed energy by the system by WSN-O and UAV-O schemes.

The total energy consumption for the complete system is shown in Figure 23 and the energy increase for both schemes for larger values of $A_{obs}$ can be observed that. This is clear since transmissions in the WSN require more energy to reach both the CHs and the selected CH sink. Additionally, as the $A_{obs}$ increased, the distance that the drone had to travel to collect the data was also higher. However, the UAV-O scheme consumed less energy for high values of $A_{obs}$. As such, it is clear that the energy depletion was higher in the system when the drone had to make several data collection stops. In this regard, saving energy in the WSN entailed a higher energy consumption in the total system. Furthermore, note that the energy consumption increased for higher values of *N*. The reason for this was that, as the number of nodes increased, more data and packet transmissions were generated in the system.

**Figure 24:**
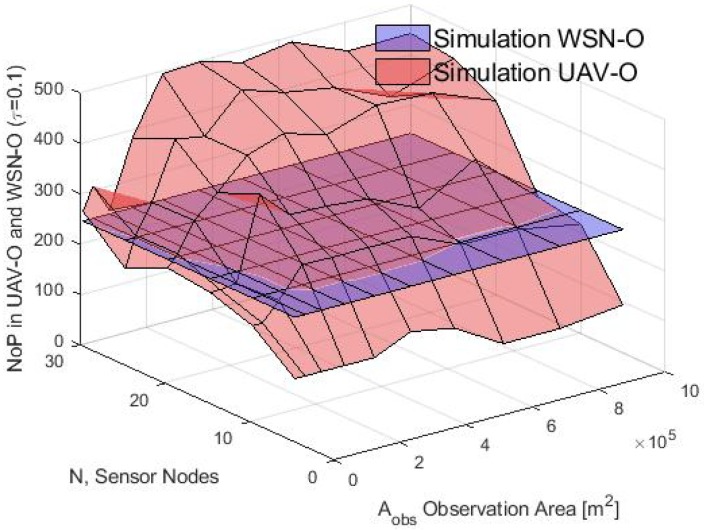
NoP for WSN-O and UAV-O scheme in simulation.

**Figure 25:**
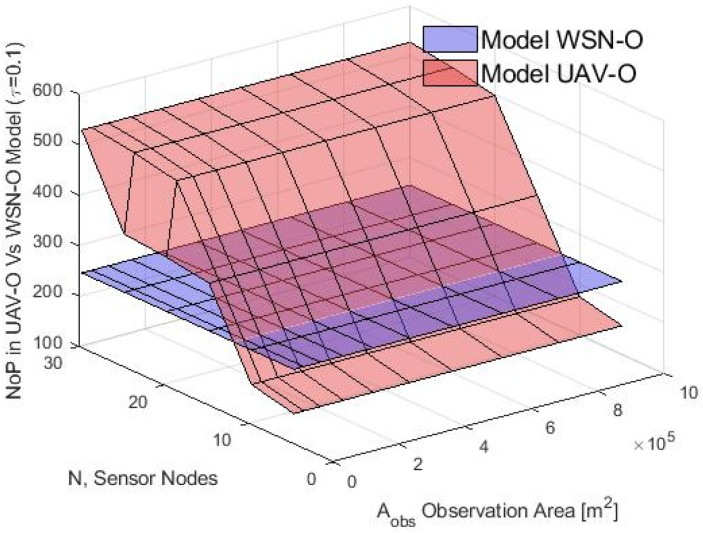
NoP for WSN-O and UAV-O scheme in model.

**Figure 26:**
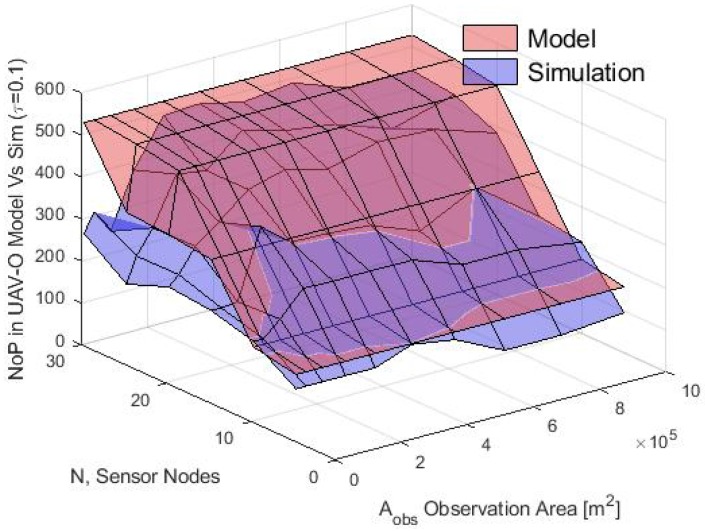
NoP for UAV-O scheme for model vs simulation.

Finally, the average buffer size in terms of NoP was calculated in the simulation and for the analytical model for both schemes and the results are shown in Figure 24 and Figure 25 respectively. In the simulation, it was observed that in the WSN-O scheme, the NoP was constant and was in fact equal to the total number of time slots of the round because the buffer size needed for the nodes was equal to the buffer size of any of the CHs. In one round, each CH received the same number of packets, irrespective of the number of nodes, CM or CHs. For instance, in this scenario, the total number of packets counted in the simulation of one round or the total packets sent from each CM to their CH, were 244 packets (analytically it was calculated as $\left\lfloor \frac{R}{P_{slot}}\right\rfloor$). In the UAV-O scheme, the NoP increased when *N* and $A_{obs}$ increased. This is because the total buffer size that the nodes need was equal to the buffer size of the unique CH_sink_ selected and hence all the packets were sent from all the CMs of the entire network depending on the total number of nodes, CMs and CHs formed then, for instance, if the $A_{obs}$ was small, the clusters could overlap between them and form only a portion of the total number of clusters sending fewer packets and if the $A_{obs}$ was longer, there was a higher probability that the total number of clusters would form and could receive the packets from all their CMs at the same time, sending more packets (but if the $A_{obs}$ was large enough, maybe it could not form any cluster because the nodes would be far apart from any of these clusters). The NoP calculated with the proposed model can be validated in Figure 26, since both graphs are similar. The slight difference between the simulation results and the mathematical model may be because the average number of time slots or CMs per cluster does not into account the exact number (but rather the average number) of CMs as in the simulation, and when the number of nodes increases, the difference between the simulation number of CMs and the average is more notorious thus for future work, we propose a more rigorous study for the calculations of the average number of CMs per cluster for the analytical model.

These results could be used as a support tool for any administrator of the WSN for remote animal monitoring with recollection data through UAV in large extension areas. For instance, if the $A_{obs}$ is about $5\times 10^{5}$ m${}^{2}$, and the administrator of the network decides to use 15 nodes, they can predict that, if they use the WSN-O scheme the CF energy that the system is going to consume is about $0.5\times 10^{5}$ EUs and almost the same in the UAV-scheme (since for both, it uses the slotted aloha protocol). However if they decide to use 30 nodes and the $A_{obs}=9\times 10$ m${}^{2}$, both schemes will consume about $6\times 10^{5}$ EUs. Then for the SS phase, the administrators could observe that with 5 nodes and $5\times 10^{5}$ m${}^{2}$ the WSN will consume about $2.5\times 10^{5}$ EUs in the WSN-O scheme, meanwhile, in the UAV-O scheme the system consumes about $1.2\times 10^{5}$ EUs, but if they deploy 30 nodes and the $A_{obs}9\times 10^{5}$ m${}^{2}$, the WSN-O will consume $2.5\times 10^{6}$ EU and the UAV-O almost $1.4\times 10^{6}$ EU, which is much lower than in the WSN-O scheme. For the UAV energy consumption, in the WSN-O scheme, the energy is depleted faster than in the UAV scheme for different $A_{obs}$ and *N*. So the network administrator can predict and decide which scheme to choose depending on how many nodes are deployed in which size of $A_{obs}$, the flight time of the UAV and the lifetime of the sensors. Also, the administrator could calculate the size of the buffer each of the sensors needs and their economic costs, depending on implemented scheme, the number of nodes used, and the monitoring area. For instance, if the $A_{obs}=5\times 10^{5}$ m${}^{2}$ and it used 30 nodes, the buffer size that the nodes need, in the WSN scheme, are 244 packets and in the UAV-O scheme, are 474 packets. Building on this, if the sensors have low capacity batteries, the team should use a UAV-O scheme for recollecting the data with the cost of acquiring nodes with greater buffer and economic costs and if the chosen UAV has a low battery capacity the administrator should choose the UAV-scheme knowing that the CH_sink_ installed on it will be expensive since it requires a high data storage capacity.

## 8. Conclusions and Future Work

In this paper, we mathematically modeled, analyzed, and studied a cluster-based WSN for animal monitoring in large extensive areas and difficult access regions with data recollection though single UAV. Two recollection schemes were proposed: (1) WSN oriented, and (2) UAV oriented. The main objective of the WSN-O method was to distribute the stored data throughout the entire network so that the UAV could visit all the CHs and in the UAV-O, the data could be stored in one single node so that the UAV only visited that node. The results derived in this work allowed one to obtain an appropriate approximation of the average energy consumption in the system for a specific number of nodes *N*, and a monitored area for each corresponding scheme. In general, the energy consumption by the system was larger in the WSN-O, but when the $A_{obs}$ was smaller, the total consumed energy was very similar for both schemes so the selection of the scheme depended on the size of the region, the number of nodes, and the nodes buffer size because if the node could not store all the data from all the CHs, the UAV-O could not be implemented. For future work, this investigation has many applications, for example a random walk model for particular animal trajectories can be developed to approximately calculate the energy cost of the WSN when the animal is going to pass inside the coverage radius of the sensor nodes and if the nodes are active or inactive, the portion of time that the animal is inside the sensor coverage radius and is active can be calculated.

## Figures and Tables

**Table 1:** Main specifications of xBee Pro 2Sc 2.4GHz.

Specification	Description
Data rate	RF 250 Kbps
Indoor/urban range	Up to 90 m
Outdoor/rf range	Up to 3200 m
Transmit power	63 mW (+18 dBm)
Reception sensitivity (1%)	−101 dBm
Serial data interface	UART, SPI
Frequency band	ISM 2.4 GHz
Antenna options	Adaptable
Dimensions	2.2 cm × 3.4 cm × 0.3 cm
Channels	16
Supply voltage	2.1 V to 3.6 V
Transmit current	33 mA
Transmit current(boost mode)	45 mA
Reception current	28 mA
Reception current (boost mode)	31 mA
Power-down current	<1 A @ 25 C
Supported topologies	Star, Mesh, Bus

**Table 2:** Physical and electrical parameters of the elected UAV.

Component	Description
Frame Size	450 mm
Frame material	Carbon fiber
Propellers	0.2032–0.254 m
Brushless motors	2212 1000 KV($\frac{rpm}{V}$)
Electronic speed controller (ESC)	30 A
ArduPilot Mega (APM)	Arducopter 2.6
Telemetry Tx/Rx	915 MHz, Omni, 100 mW
Lipo BAttery	2400–4000 mAh, 12 V
GPS	6 M (−162 dBm sens)
Approx. total weight	700–800 g
Main inertia moments	0.026705 kg·m${}^{2}$
